# The Bezold–Jarisch reflex: Hubris and (n)emesis in the clinical assessment of acute inferior wall myocardial infarction

**DOI:** 10.1113/EP092587

**Published:** 2025-03-03

**Authors:** Ronan M. G. Berg, Reza Jabbari

**Affiliations:** ^1^ Centre for Physical Activity Research Copenhagen University Hospital – Rigshospitalet Copenhagen Denmark; ^2^ Department of Clinical Physiology and Nuclear Medicine Copenhagen University Hospital – Rigshospitalet Copenhagen Denmark; ^3^ Department of Clinical Medicine, Faculty of Health and Medical Sciences University of Copenhagen Copenhagen Denmark; ^4^ Neurovascular Research Laboratory, Faculty of Life Sciences and Education University of South Wales Pontypridd UK; ^5^ Department of Cardiology Copenhagen University Hospital – Rigshospitalet Copenhagen Denmark

**Keywords:** bradycardia, cardiology, cardiovascular physiology, electrocardiogram, history, ischaemia, myocardium, reflex

1

Recently, the former editor‐in‐chief of *Experimental Physiology*, Professor Mike Tipton, conceived the new ‘Lived Experience’ section of the journal and introduced it with his own account of completing the 2024 Ironman event in Klagenfurt, Austria, to convey and reflect on the many lessons that can be learned from such an experience regarding integrative physiology (Tipton, 2025). In this issue, Dr Richard Godfrey from Brunel University London, also an active athlete, follows up with his compelling first‐person account of atypical angina that ultimately progressed to an acute inferior wall myocardial infarction caused by right coronary artery thrombosis owing to an unrecognized underlying antiphospholipid antibody syndrome (Godfrey, 2025). The author's narrative of his personal journey during the recovery process, based on this autoethnography and his experience with cardiac rehabilitation, forms the basis of his hypothesis that high‐intensity interval training might serve as a potential promoter of cardiac regeneration. Furthermore, the narrative sheds light on the underlying pathophysiology of myocardial blood flow regulation and associated cardiovascular reflexes.

Curiously, in Dr Godfrey's case, the presenting symptoms were dominated by gastrointestinal issues, particularly emesis, i.e., nausea and vomiting. Such symptoms are often observed as accompanying features of angina pectoris, recognized as far back as by Sir William Osler (1849–1919), who described them as a grave sign, often heralding a fatal outcome (Osler, 1910). But rarely, as in this case, they present as the dominant symptoms.

Emesis is often stated to signify the site of myocardial ischaemia, specifically as a sign of inferior wall myocardial ischaemia, sometimes even referred to as ‘cardiac vomiting’ (Sleight, 1981). In Denmark, this has led to the triple‐B mnemonic, ‘bræk, blok & brady’, referring to the triad of vomiting, atrioventricular block and sinus bradycardia. The mechanistic link between nausea and vomiting and inferior wall myocardial ischaemia is a ‘classic’ among cardiovascular reflexes: the so‐called Bezold–Jarisch reflex (Aviado & Aviado, 2001; Mark, 1983). This reflex is named after Albert von Bezold (1836–1868) and Adolf Jarisch Jr (1891–1965), of which von Bezold served as an assistant to Emil Du Bois‐Reymond (1818–1896). Of note, it was Du Bois‐Reymond who, along with his friends Hermann von Helmholtz (1821–1894), Ernst von Brücke (1819–1892) and Carl Ludwig (1816–1895), who formed the renowned ‘1847 group’, which marked a pivotal transition in physiology, steering it away from its roots in natural philosophy to an empirically driven discipline, aiming to elucidate the processes of life and the mechanisms of disease through formal experimentation on the basis of physics and chemistry. This shift established physiology as a basic science in its own right, with an experimental approach that forms the scientific foundation of our field to this day.

The Bezold–Jarisch reflex is an inhibitory reflex originating in cardiac sensory receptors with vagal afferents, which are influenced by either chemical or mechanical stimuli (Aviado & Aviado, 2001). Such noxious stimuli detected in the cardiac ventricles are transmitted via vagal afferent C fibres to the nucleus tractus solitarii. This triggers an acute increase in vagal activity and sympathetic withdrawal, leading to systemic hypotension through bradycardia and peripheral vasodilatation, in addition to hypopnoea through the effects of central inhibitory pathways on the respiratory network in the brainstem. Albert von Bezold and Ludwig Hirt (1844–1907) first demonstrated the reflex in 1867 through intravenous injection of an alkaloidal extract of green false helleborine (*Veratrum viride*) or mistletoe (*Viscum album*) in rabbits (von Bezold & Hirt, 1867). Adolf Jarisch Jr and colleagues re‐examined the reflex in cats in the 1930s, showing that it originated in the heart rather than the great vessels and that it was mediated by the vagus nerve and relayed in the nucleus tractus solitarii (Jarisch Jr, 1940; Jarisch Jr & Richter, 1939).

Metabolites generated in ischaemic and/or infarcted myocardium are among the stimuli that trigger the Bezold–Jarisch reflex. In this context, the reflex is likely to provide vagal cardioprotection to myocardial areas at risk (Gelpi, 2016), by reducing myocardial oxidative metabolism and increasing the duration of diastole, when myocardial perfusion occurs, through bradycardia. The link to inferior wall myocardial ischaemia is provided by a preferential distribution of vagal receptors on the inferoposterior left ventricular wall (Felder & Thames, 1979; Thames et al., 1978; Walker et al., 1978), which is the typical right coronary artery territory. This anatomical predisposition explains why the reflex is more commonly triggered during inferior rather than anterior wall ischaemia. Although not formally defined as part of the Bezold–Jarisch reflex, it has been shown to be closely associated with emesis, because stimulation of inhibitory cardiac receptors with vagal afferent fibres during coronary occlusion produces reflex gastric dilatation and retching (Abrahammson & Thoren, 1973), a response that is more pronounced during stimulation of cardiac receptors in the inferior compared with the anterior ventricular wall (Johannsen et al., 1981).

On the basis of the studies that were conducted mainly in the 1970s, previous anecdotal claims that emesis is specifically a sign of inferior wall myocardial infarction seemed entirely plausible (see, e.g., James, 1968), and this was further substantiated following the publication of an observational study of 62 patients with a diagnosis of either anterior or inferior wall acute myocardial infarction (Ahmed et al., 1978). The study reported that 27% of patients with anterior wall myocardial infarction complained of nausea and vomiting, in comparison to 69% of patients with inferior wall myocardial infarction (Ahmed et al., 1978). Soon, this finding gained widespread acceptance (Mark, 1983; Sleight, 1981). Indeed, the study has continued to be cited as evidence supporting the claim, despite being corroborated by only one additional study (Culić et al., 2001). Over the >45 years that have passed since then, other studies have failed to replicate this finding (Fuller et al., 2009; Herlihy et al., 1987; Ingram et al., 1980). Indeed, other factors, such as the size of the infarction, have been highlighted as potentially more relevant triggers of Bezold–Jarisch‐associated emesis.

Although the link between emesis and inferior localization of myocardial ischaemia, as also evident in the paper describing Dr Richard Godfrey's case, fits beautifully from a mechanistic perspective, the complexity of bodily functions in a real‐world setting means that such a concept cannot necessarily be translated uncritically to clinical practice. The historical persistence of this idea, despite compelling evidence to suggest otherwise, might reflect the ‘translational gap’ between mechanistic sciences and clinical practice. This gap is likely to be one reason why evidence‐based medicine currently places mechanistic evidence at the very bottom of the evidence hierarchy for informing clinical decision‐making (Berg et al., 2025), which is a paradox given that Carl Ludwig, in his letters to Du Bois‐Reymond, defined the purpose of physiology as an independent basic science as ‘to serve the clinics’. However, this purpose remains valid. Mechanistic principles and concepts, even when not directly translatable, provide an essential framework for understanding and advancing clinical medicine. As the motto of *The Physiological Society* asserts, ‘Physiology is the science of life’, encompassing the processes of living organisms, from the cellular to the applied level. Physiology underpins everyday life, adaptation to extreme environments, athletic performance and clinical practice, a breadth perfectly embodied in the concept of ‘Lived Experience’. Moreover, physiology is not only a science of life but also a living science, self‐correcting and iterative in nature. Our understanding of the interplay between known mechanisms, health and disease evolves continuously through models derived from experimentation, observations and experiences in clinical settings. The history of the Bezold–Jarisch reflex is testament to that, as is Dr Godfrey's story.

## AUTHOR CONTRIBUTIONS

Ronan M. G. Berg: Conception, first draft, revisions. Reza Jabbari: Revisions. Both authors approved the final version of the manuscript and agree to be accountable for all aspects of the work in ensuring that questions related to the accuracy or integrity of any part of the work are appropriately investigated and resolved. Both persons designated as authors qualify for authorship, and all those who qualify for authorship are listed.

## CONFLICT OF INTEREST

None declared.
